# Supplementary material for: Field-based species identification of closely-related plants using real-time nanopore sequencing
Source: Sci Rep. 2017 Aug 21;7:8345. doi: 10.1038/s41598-017-08461-5 (PMC5566789; doi:10.1038/s41598-017-08461-5)
Supplement: Supplementary file 1 — Supplementary Information [file 41598_2017_8461_MOESM1_ESM.pdf]

Title:
Field-based species identification of closely-related plants using real-time nanopore sequencing.

Authors:
Joe Parker[1]*, Dion Devey[1], Andrew J. Helmstetter[1], Tim Wilkinson[1] & Alexander S.T. Papadopulos[1,2]*

[1]Jodrell Laboratory, Royal Botanic Gardens, Kew, Richmond, Surrey UK. TW9 3AB
[2]Molecular Ecology and Fisheries Genetics Laboratory, Environment Centre Wales, School of Biological Sciences, Bangor University, Bangor, UK, LL57 2UW.

*Correspondence to a.papadopulos@kew.org and joe.parker@kew.org

Keywords:
Nanopore, RTnS, MinION, onsite DNA sequencing, phylogenomics

Supplementary Information

Contents:
Extended Data Figures
Extended Data Tables
Supplementary Methods
Supplementary Tables
Supplementary Discussion
Supplementary Notes

Extended Data

a

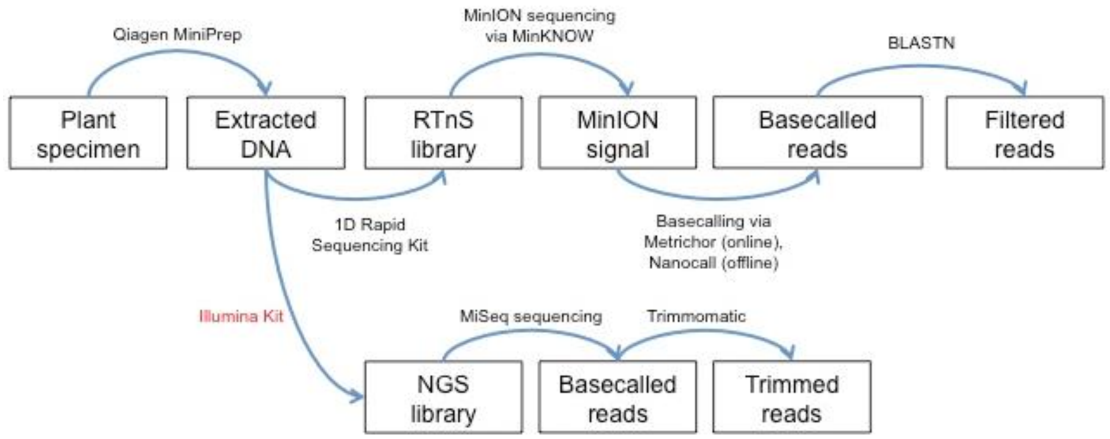

b

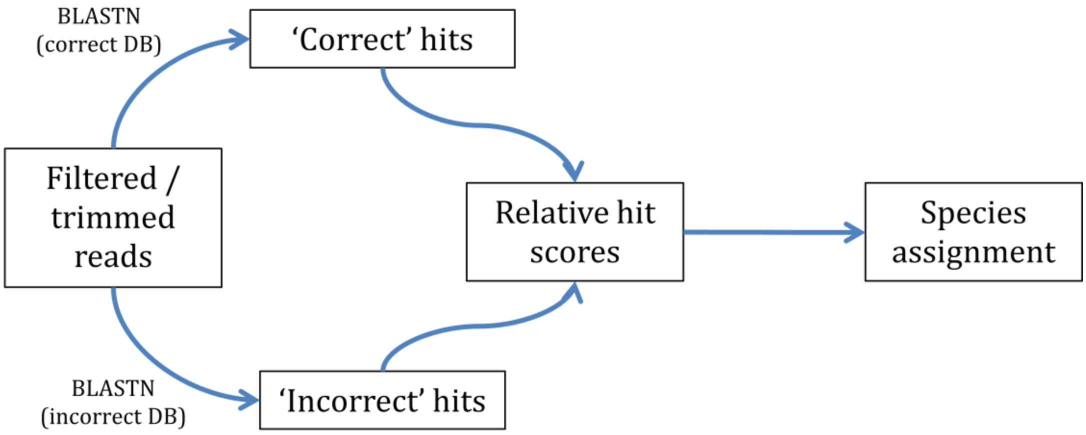

c

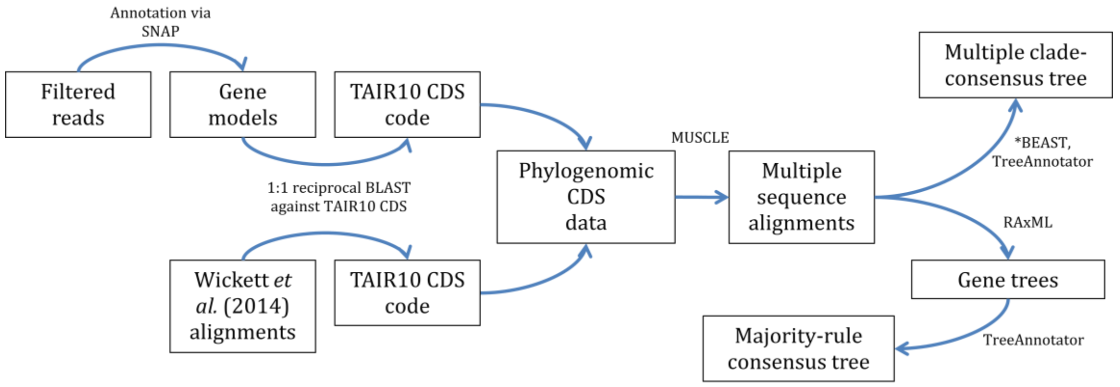

Extended Data Figure 1 | Schematic of experimental workflows. a, Sampling-to-sequencing workflow. **b,** Sample identification workflow via BLASTN. **c,** Outline for direct annotation of raw RTnS reads followed by phylogenomic inference. See Methods for details.

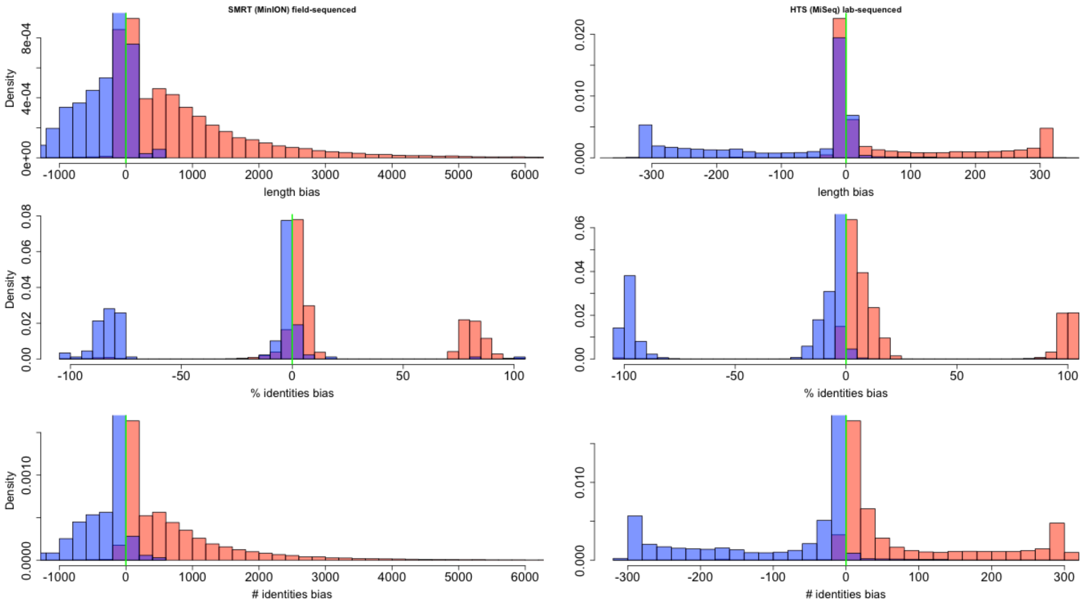

Extended Data Figure 2 | Distribution of difference statistics in BLASTN comparisons for species ID. Empirical distribution of test statistics for each-way congeneric sample ID (binary classification) using BLASTN evaluated for RTnS MinION (*left column*) and NGS MiSeq (*right column*) platforms. Difference (test) statistics were calculated for each alignment as (true positive (TP) score – false positive (FP) score) for each of: alignment length; % identities; and number of identities. Reads sequenced from *A. thaliana* samples (comprising nominal true positives and false-positives (contaminants) are shown in red; reads from *A. lyrata* samples (nominal true negatives) shown in blue. 'True' and 'false' distributions' overlap is small, while alignment length and number of identities' distributions are both unimodal, showing a simple cutoff-based classifier should perform well to discriminate between 'true' and 'false' cases.

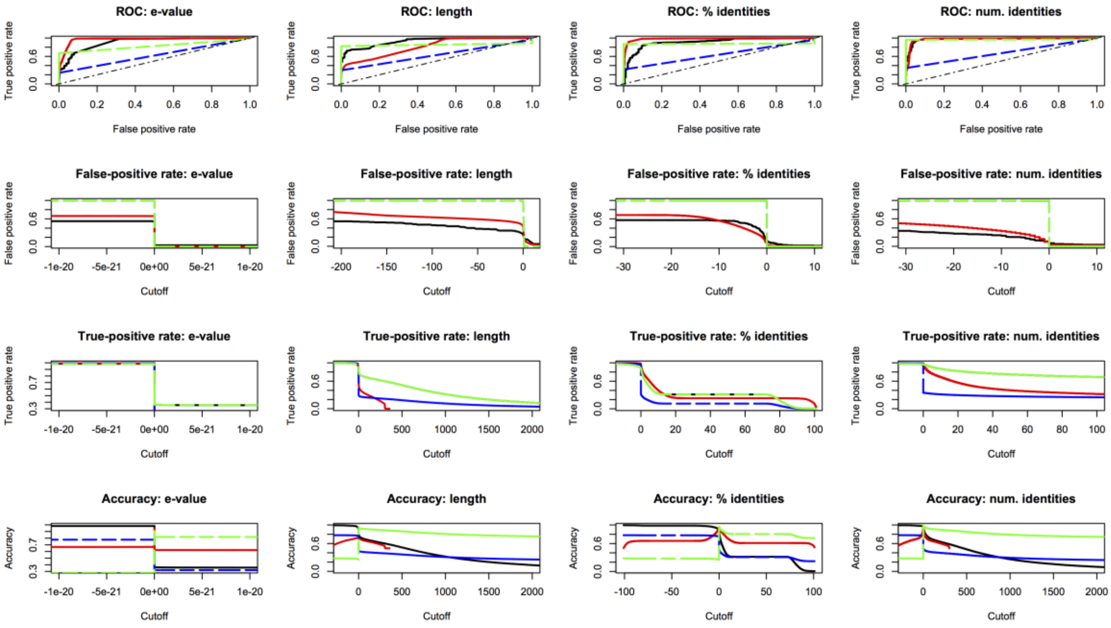

Extended Data Figure 3 | Performance of difference statistics in BLASTN comparisons for species ID. Performance of test statistics for each-way congeneric sample ID (binary classification) using BLASTN evaluated for MinION (*black*) and MiSeq (*red*) platforms. Difference (test) statistics were calculated amongst reads matching both databases for each alignment as (true positive (TP) score – false positive (FP) score) for each of e-value, length, % identities and number of identities. Reads matching only one or neither database were additionally included with either 'all-false' encoding (dashed green line) or mixed false and true encoding depending on sample origin (blue line; see Methods and Supplementary Information for details). RTnS reads' true- and false-positive rates are comparable to, and in some cases better than, NGS reads' performance; while the longer length of RTnS reads permits the use of high thresholds where greater confidence is desired (perhaps in the case of very closely related specimens). *Top row:* true-positive (TP) vs false-positive (FP) rate; classical receiver operating curve. *Second row:* FP rate with varying test statistic threshold. *Third row:* TP rate with varying test statistic threshold. *Bottom row:* Accuracy with varying test statistic threshold. Accuracy estimated as (TP+TN / (P + N)). *Columns (L-R):* Difference statistics for *e*-value, total alignment length, % identities, and number of identities, respectively.

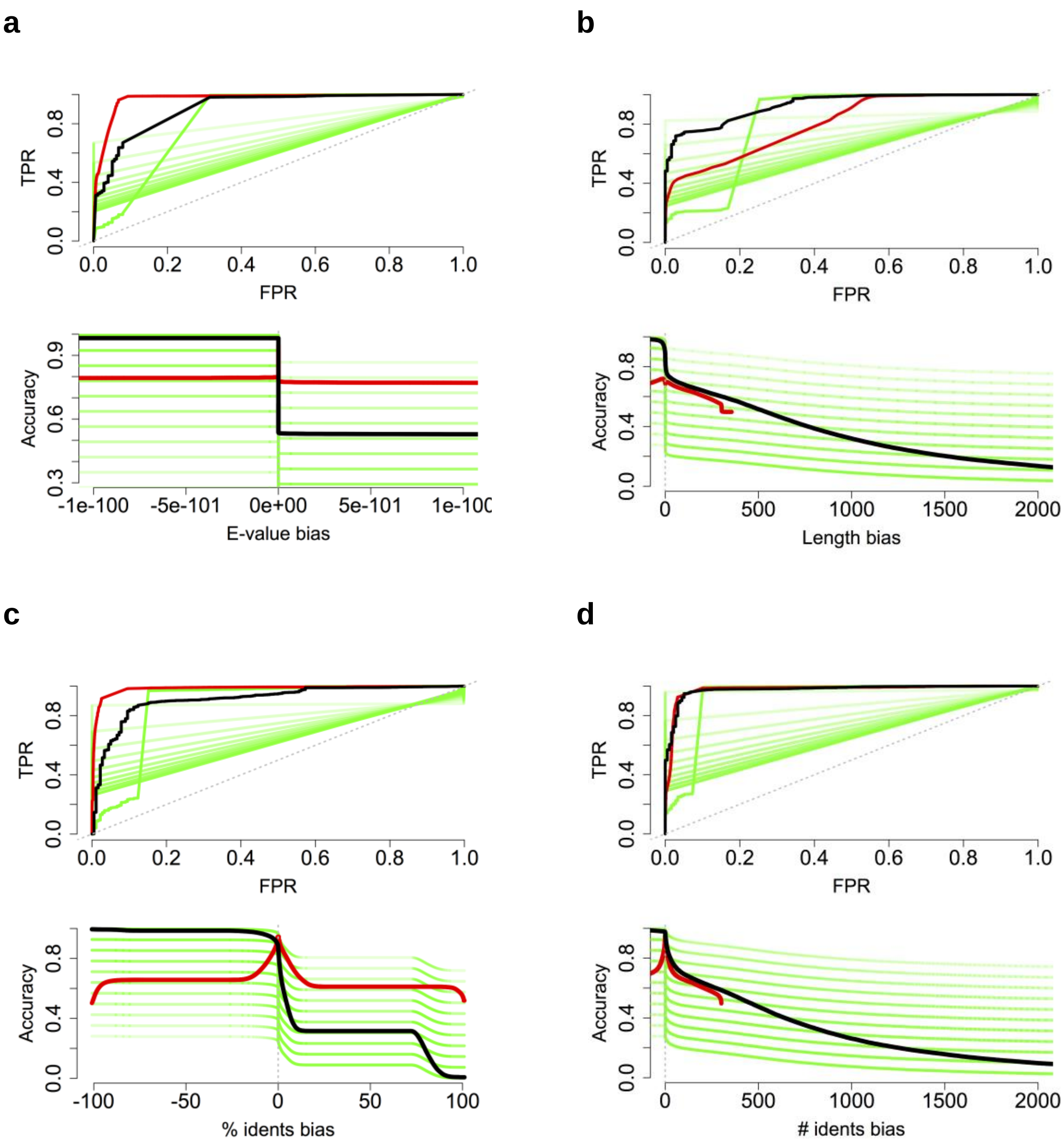

Extended Data Figure 4 | Modelling potential effect of incorrectly estimated TN/FN proportions. Red and black lines show empirically estimated statistical performance for species ID via BLASTN comparison of HTS and RTnS reads respectively (as for Extended Data Figure 3; see Methods for details). Reads that produced no hits to either database might represent false negatives (sequencing error, or genomic regions not represented in the reference genome BLAST databases) or true negatives (sequencing contaminants and sequencer noise). These nonmatching reads were to reflect 'true negative':'false negative' ('TN:FN') mixtures in 10% increments shown from light to dark green shading. Plots (**a-d**) show results for bias statistics in *E*-value; total alignment length; % identities; and total alignment identities, respectively. Extreme TN:FN mixtures still display adequate true-positive vs. false-positive rates; empirical data is approximated by the 30% TN:FN mixture, approximately reflecting the proportions of *A. lyrata* to *A. thaliana* nonmatching reads in the dataset.

a
b
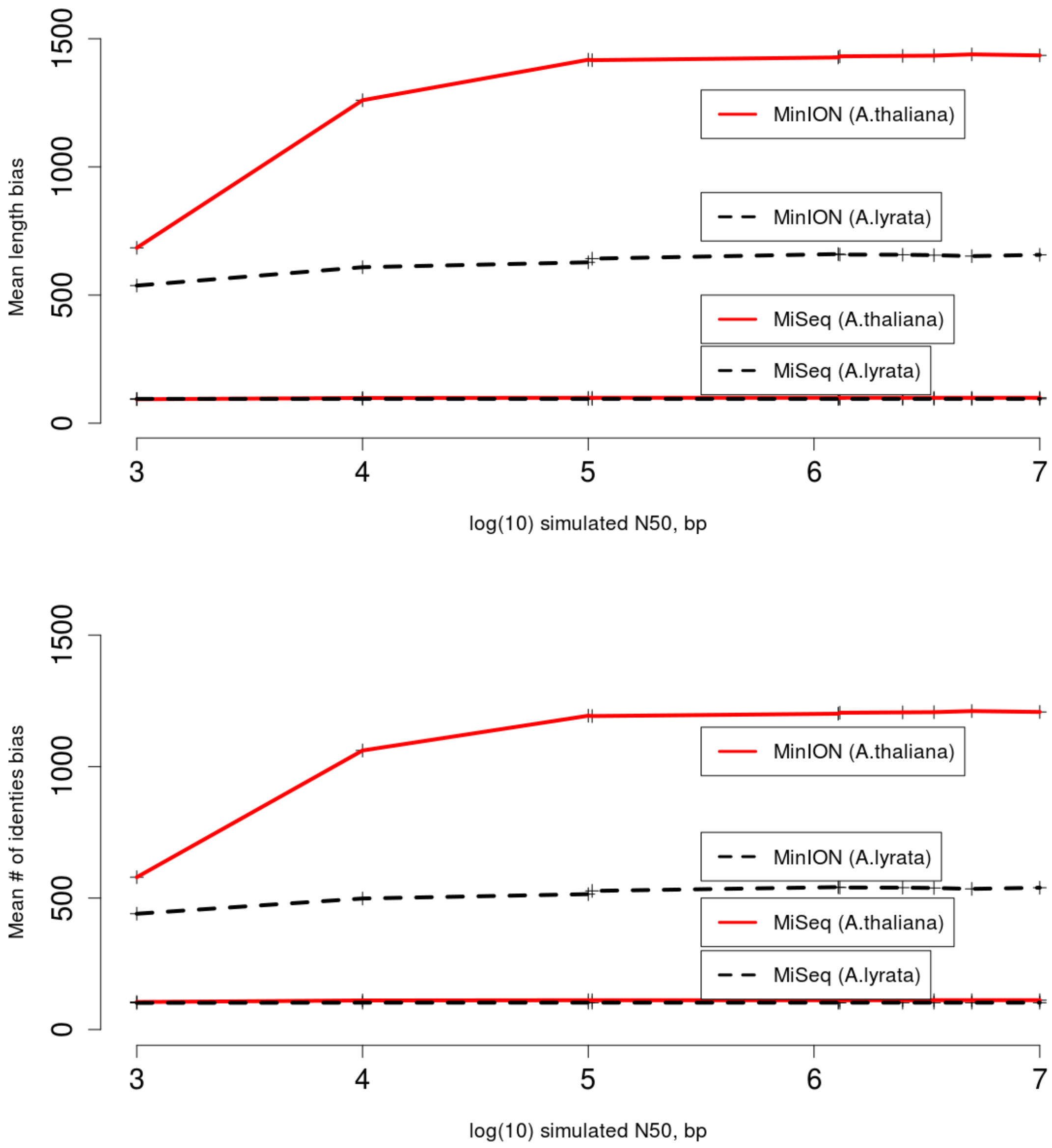

Extended Data Figure 5 | Simulations of the effect of genome contiguity (N50) on species identification. Differences in (a) total alignment lengths (ΔL_T) and (b) number of identities (ΔL_I) at various N50 values. Decreasing genome contiguity is accompanied by decreases in the statistics used for species identification. Even at low contiguity both statistics provide confident species assignment using long read data.

a

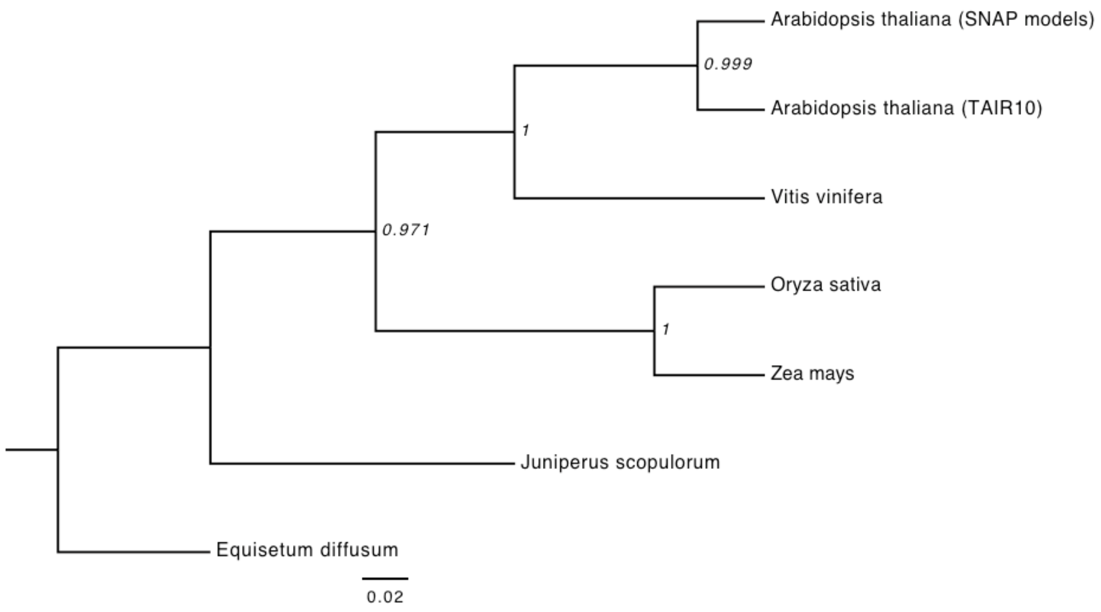

b

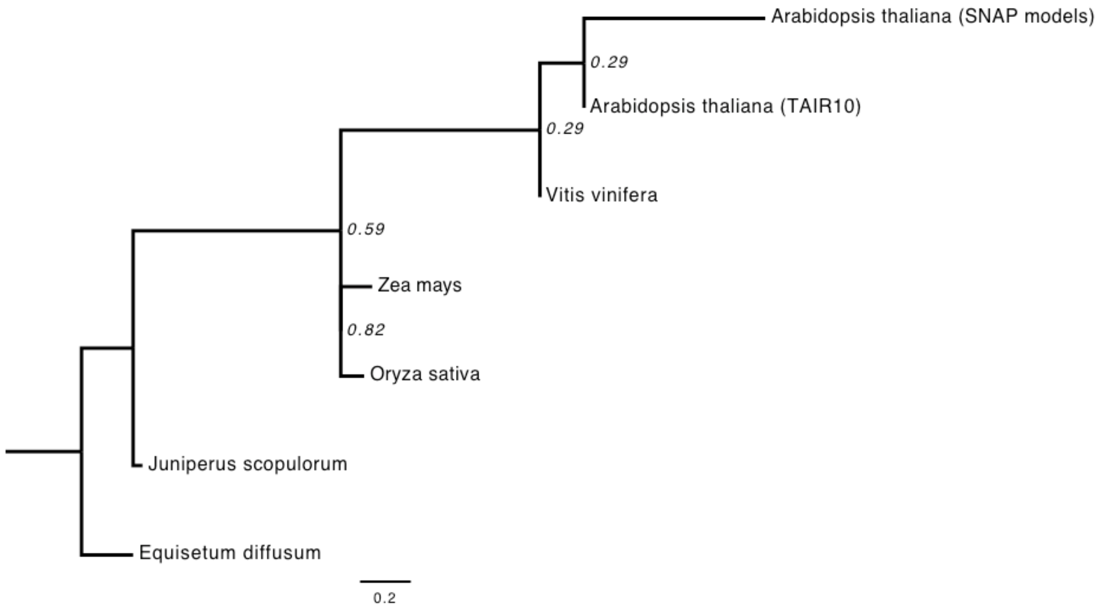

Extended Data Figure 6 | Phylogeny of species spanning major plant groups. Putative coding sequences recovered from single, unassembled raw RTnS reads using SNAP *ab initio* gene prediction could be aligned to existing phylogenomic data from other taxa and used to infer a phylogeny consistent with accepted plant relationships. **a,** Multispecies coalescent species tree inferred from 18 gene trees (genes predicted directly from raw nanopore reads). Inferred using multispecies coalescent implemented in BEAST 2.4.4; **b,** consensus species tree inferred by majority-rule from 18 gene trees, inferred with RAxML 7.2.8.

Extended Data Table 1 | List of field-sequencing equipment.

Item / model	Quantity	Supplier
Laptops, Portege R830-1DZ	2	Toshiba
Laptop, MacBook Pro	1	Apple
Portable firewire HDD, 1Tb	2	LaCie
Portable AC generator, IMPAX IM800I 700W	1	ScrewFix
Thermal control textiles (socks) for MinION	Pair	ScrewFix
AC extension plugs, 4-way, 3-20M	3	Argos
Portable folding tables	2	Argos
Weather meter, Kestrel 5500	1	KestrelMeters
Fluorometer, Quantus	1	Promega
Water bath, GD100	1	Grant
Microcentrifuge, 5415C	1	Eppendorf
Glass thermometers, 300mm, range 263.3-383.3ºK	2	
Arduino Uno	1	Maplins
Themal transducers, LM335AZ	4	RS Components
Polystyrene thermal control boxes, various sizes	3	
Freezer coolpacks	14	
Pipettes, Gilson:	-	
P2	1	ThermoFischer
P20	1	ThermoFischer
P200	1	ThermoFischer
P1000	1	ThermoFischer
Waste containers	2	
Laboratory consumables:	-	
PCR tubes, thin-walled	Box	ThermoFischer
Eppendorf tubes, 1.5ml DNA lo-bind	Box	ThermoFischer
Eppendorf tubes, 2.0ml DNA lo-bind	Box	ThermoFischer
Plastic pestles	20	
Sterilized sand	5g	
Reagent tubes, screw-top, 50ml	10	ThermoFischer

Extended Data Table 2 | Performance of MinION sequencing runs

Species	*A. thaliana*	*A. thaliana*	*A. lyrata*	*A. lyrata*
**Reaction chemistry**	R7.3, 1D	R9, 1D	R7.3, 1D	R9, 1D
**Run IDs**	2507 2126 3637	5913 2144 0509	4901 1842 1201	1222 5458 1958 0824 2912 5201
**Start time**	18/05/2016 21:36	18/05/2016 18:21	19/05/2016 16:10	19/05/2016 16:39:20
**Latest read***	19/05/2016 15:17:00	19/05/2016 15:18:39	24/05/2016 01:14	22/05/2016 07:08:56
**Disc space (raw)**	2.9Gb	106.8Gb	3.0Gb	35.1Gb
**Metrichor ID**	115360	115459	115375	115432
**# reads**	4,152	92,693	2,387	23,452
**Yield (bp):**				
total	7,351,585	233,244,147	16,092,487	46,118,754
mean	1,771	2,516	6,742	1,967
median	586	1,396	120	305
maximum	434,377	170,598	1,114,970	177,310
N25	19,244	9,651	574,112	24,254
N50	4,771	4,410	309,034	7,926
N75	2,041	2,121	62,360	3,374

Data produced with field-extracted and field-sequenced in conditions ranging from 6-14ºC and up to 100% humidity, producing genome-scale sequence data despite several pauses in sequencing to dismantle, relocate, and reassemble equipment. The two latest sequencing runs (*A. lyrata* samples) performed markedly worse than the two earliest runs (*A. thaliana)*, possibly due to the impact of storage temperature (passively controlled, and steadily rising over the week) on reagent performance. Yield summary statistics refer to untrimmed raw reads, including phage-lambda experimental control in the case of *A. thaliana* R9 data (filtered from subsequent steps). See Methods for details. Note: *Final *A. lyrata* sequencing phase performed in laboratory owing to time constraints on-site.

Extended Data Table 3 | Statistics of reference genomes used

**Species**	***Arabidopsis lyrata ssp. petraea***	***A. lyrata***	***A. thaliana***
**Version**	1.0	1.0	TAIR10
**Date accessed**	25/05/2016	17/05/2016	17/05/2016
**Accession / ID**	NCBI: assembly= GCA_000524985.1	NCBI: genome=493 genome_assembly=29434	NCBI: genome=4 genome_assembly=22492
**Total assembly length**	202,972,003	206,667,935	119,667,750
**Total gap length**	20,456,163	22,960,134	185,644
**Number of scaffolds**	281,536	695	7
**Scaffold N50**	7,848	24,464,547	23,459,830
**Scaffold L50**	6,426	4	3
**Number of contigs**	369,168	3,645	102
**Contig N50**	2,321	227,391	11,194,537
**Contig L50**	16,831	247	5
**Total chromosomes & plasmids**	0	0	7

A. thaliana TAIR10 release is considerably more complete than either of the draft *A. lyrata* assemblies.

Extended Data Table 4 | Sample identification via BLASTN

Sample	*A. thaliana*	*A.lyrata*	*A. thaliana*	*A.lyrata*
**"TRUE" database**	*A. thaliana*	*A.lyrata* combined*	*A. thaliana*	*A.lyrata* combined*
**"FALSE" database**	*A.lyrata* combined*	*A. thaliana*	*A.lyrata* combined*	*A. thaliana*
**Source data**	ONT 1D	ONT 1D	MiSeq	MiSeq
**# Reads, total**	91,715	25,839	9,476,598	9,659,489
**# Reads with BLASTN hits:**				
1-way TRUE †	10,322	76	2,140,403	2,907,921
1-way FALSE ‡	378	2	53,056	24,329
2-way BOTH §	22,386	101	7,098,032	6,256,969
0-way ZERO ¶	58,629	25,660	185,107	470,270
**Proportion of reads:**				
1-way TRUE	*0.113*	*0.003*	*0.226*	*0.301*
1-way FALSE	*0.004*	*0.000*	*0.006*	*0.003*
2-way BOTH	*0.244*	*0.004*	*0.749*	*0.648*
0-way ZERO	*0.639*	*0.993*	*0.020*	*0.049*
**Biases #:**				
Mean length	1,323.87	698.25	83.61	108.99
Mean identities	1,115	575	96	117
Mean % identities	37.97	61.41	30.16	42.38
Mean E*-values*	4.80E-07	1.05E-04	1.07E-08	3.52E-09

Individual RTnS and NGS reads were aligned to *A. thaliana* and *A. lyrata* databases (designated TRUE or FALSE depending on sample origin) with BLASTN, keeping the single best-hit alignment for each database. More '1-way' (only one database matched) hits to TRUE than FALSE databases accumulated in all sample / technology combinations. Amongst '2-way' hits, positive differences in the metrics were consistent with correct sample identification. For RTnS reads differences between TRUE and FALSE hits were considerably larger than amongst HTS reads (by an order of magnitude for length or number of identities), showing that confident identification could be made with fewer RTnS reads. Notes: **A. lyrata* and *A. lyrata ssp. petraea* databases combined, see Methods; †Total number of reads matching only conspecific database ('true-positives'); ‡Total number of reads matching only pairwise-compared database ('false-positives' in the case of a mixed /multiplexed sample, or 'false-negatives' in the case of a single sample); §Total number of reads matching both databases; ¶Total number of reads with no hits in either comparison, e.g. 'false-negatives'; #Difference statistics for each query read calculated as (score conspecific comparison – score congener comparison), for BLASTN alignment length, alignment identities, alignment % identities and *E*-value; ☆Mean bias across all reads.

Extended Data Table 5 | Performance of *de novo* genome assembly

**Species**	***Arabidopsis thaliana***		***A. lyrata ssp. petraea***	
**Data**	**MiSeq**	**MiSeq + MinION**	**MiSeq**	**MiSeq + MinION**
**Assembler ***	**Abyss**	**hybridSPAdes**	**Abyss**	**hybridSPAdes**
Illumina MiSeq NGS reads, 300bp paired-end	8,033,488	8,033,488	8,143,010	8,143,010
NGS total yield	2,418,079,888	2,418,079,888	2,451,046,010	2,451,046,010
Oxford Nanopore MinION RTnS reads, R7.3 + R9, N50 ~ 4,410bp	n/a	96,845	n/a	25,839
RTnS reads total yield	n/a	240,597,532	n/a	62,211,241
# contigs	24,999	10,644	37,568	85,599
Largest contig	89,717	413,462	101,114	38,313
Total length	106,455,313	119,031,857	151,562,895	117,256,694
Reference length	*119,667,750* †	*119,667,750*	*183,707,801*‡	*183,707,801*
GC content (%)	35.97	36.20	36.16	36.55
N50 §	7,853	48,730	9,605	1,686
Unaligned length	7,121,882	6,737,059	36,669,847	35,287,390
Genome fraction (%)	82.0	88.7	53.4	43.7
Duplication ratio	1.01	1.058	1.17	1.02
# N's per 100 kbp	1.72	5.41	0.22	7.09
# mismatches / 100 kbp	518	588	1,297	1,097
# indels / 100 kbp	120	130	334	271
Largest alignment	76,935	264,039	44,515	17,201
Total aligned length	98,382,255	108,086,256	100,502,092	80,814,492
Coding loci completeness ¶*:*				
# genes, 'complete'	**219**	**245**	n/a	n/a
% genes, 'complete'	88.31%	98.79%		
# genes 'partial'	**238**	**246**	n/a	n/a
% genes, 'partial'	95.97%	99.19%		

Field-extracted DNA material was of sufficient quality to enable a *de novo* assembly with lab-sequenced NGS data. Furthermore, field-sequenced RTnS reads considerably augmented the NGS data in hybrid assembly, greatly improving contiguity and estimated coding loci coverage substantially without substantially raising basewise error rates. Notes: **de novo* genome assemblies used either lab-sequenced short-read HTS data only (Abyss) or both HTS and field-sequenced RTnS datasets (Hybrid-SPAdes). †TAIR10 release. ‡INSDC: *A. lyrata*: ADBK00000000.1 (Hu *et al.,* 2011); *A. lyrata ssp. petraea*: BASP00000000.1 (Akama *et al.*, 2014). §Assembly statistics calculated using QUAST 4.0. ¶Approximate completeness of coding loci assessed via CEGMA. See Methods for details.

Supplementary Methods

Treatment of unobserved BLAST hit data. For reads matching a single database only, a T or F assignment was made and difference statistic calculated by masking unobserved alignment scores as 'extreme' (-1 each for length and % identities, 999 for *e*-value). Reads that produced no hits to either database might represent false negatives (sequencing error, or genomic regions not represented in the reference genome BLAST databases) or true negatives (sequencing contaminants and sequencer noise). To model the effect of including these nonmatching reads, dummy rows (one for each nonmatching read RTnS read: 58,629 from the *A. thaliana* experiment, and 25,660 from the *A. lyrata* experiment) were coded with ‘false’ labels and difference statistic values of zero. Proportions of these dummy reads were recoded with ‘true’ labels from 0-100% in 10% increments and classifier statistical performance was recalculated and plotted. These are shown Extended Data Figure 4 in green; where TN:FN mixtures from 0-100% TN by 10% increments shown from light to dark green shading.

Supplementary Tables

MinION read	Wickett *et al.* (2014) alignment	BLASTN assignment		
		Length	% idents	*E*-value
2bbfc883-b540-40d0-a939-25d7ef4de290_Basecall_Alignment_templat-snap.1|AT2G38440|no_kog_value	FNA.7457	1841	78.707	0
969907bc-ed37-41c0-a4aa-38f8f59f574e_Basecall_Alignment_templat-snap.1|AT1G70070|no_kog_value	FNA.6290	1820	80.22	0
aa7b4f85-8a7f-49a6-94f5-21511445c454_Basecall_Alignment_templat-snap.2|AT5G55540|no_kog_value	FNA.6857	1473	85.064	0
78aa4fe7-e644-48e4-9e45-6c13c876ef36_Basecall_Alignment_templat-snap.1|AT5G25070|no_kog_value	FNA.8121	1473	83.707	0
afd5fe61-8b9f-41fd-849e-fe66347628e5_Basecall_Alignment_templat-snap.1|AT2G05320|no_kog_value	FNA.7510	1327	77.995	0
6399de40-39c7-4a3f-987d-af2560995ff4_Basecall_Alignment_templat-snap.2|AT3G50370|no_kog_value	FNA.4982	1299	86.143	0
b0238c1e-fa62-4eab-bfb1-33771be23b40_Basecall_Alignment_templat-snap.4|AT5G54880|no_kog_value	FNA.7601	1219	79.327	0
b7dbb53d-8730-41ca-8859-fb2fce9dd3a2_Basecall_Alignment_templat-snap.1|AT5G62030|no_kog_value	FNA.6807	1196	86.873	0
a1bef598-15d2-4c0e-bc84-96a7535d4d5c_Basecall_Alignment_templat-snap.2|AT5G41020|no_kog_value	FNA.5991	1176	88.095	0
ae7825fc-6fe7-4c98-83cb-4b399b05dd49_Basecall_Alignment_templat-snap.3|AT5G24260|no_kog_value	FNA.5758	1118	87.478	0
959fcf39-5c60-488a-9cfb-1d1292104451_Basecall_Alignment_templat-snap.1|AT4G35870|no_kog_value	FNA.7223	1101	89.101	0
14886dbf-88fc-4b52-acc3-9dd88883fe78_Basecall_Alignment_templat-snap.1|AT5G16850|no_kog_value	FNA.7056	1068	85.393	0
9be32191-63a2-4653-9f48-3046010253b8_Basecall_Alignment_templat-snap.1|ATMG01360|no_kog_value	FNA.6476	990	86.768	0
ed0952ad-4353-4f49-87a5-0aebcd47d6bb_Basecall_Alignment_templat-snap.1|AT1G07970|no_kog_value	FNA.7418	940	80.638	0
282b79da-8c79-4438-bf70-ccf51afe1ad8_Basecall_Alignment_templat-snap.2|AT5G42950|no_kog_value	FNA.6032	909	83.278	0
73cda463-e9ba-4410-9520-46a26748e332_Basecall_Alignment_templat-snap.3|AT5G66840|no_kog_value	FNA.4932	864	83.449	0
7a9a13ac-d903-43ab-ab42-18e86fbc1874_Basecall_Alignment_templat-snap.3|AT4G20060|no_kog_value	FNA.6092	763	84.273	0
8b3c34a2-8f0f-424a-9d57-7d12e8575f81_Basecall_Alignment_templat-snap.1|AT1G12800|no_kog_value	FNA.6660	747	85.274	0
5171e626-5821-4c4a-8b0b-2bd584d82b38_Basecall_Alignment_templat-snap.2|AT3G08800|no_kog_value	FNA.7511	686	88.047	0
591fe8b1-db83-4eff-a1d0-2653692b0b62_Basecall_Alignment_templat-snap.5|AT4G14180|no_kog_value	FNA.7300	667	85.907	0
5d922a41-af38-4697-8467-fa4fa2836e5f_Basecall_Alignment_templat-snap.1|AT1G23180|no_kog_value	FNA.5750	666	85.886	0

MinION read	Wickett *et al.* (2014) alignment	BLASTN assignment		
		Length	% idents	*E*-value
6bdb48cb-40c5-43af-b18f-5140ebcf8256_Basecall_Alignment_templat-snap.1|AT2G35790|no_kog_value	FNA.7147	647	86.708	0
85a6e673-d25c-4d88-89ad-40c0edb9a5ee_Basecall_Alignment_templat-snap.3|AT1G21710|no_kog_value	FNA.6635	645	93.953	0
cb359e33-b388-40d1-8cda-e3d730373663_Basecall_Alignment_templat-snap.2|AT4G00450|no_kog_value	FNA.5763	638	89.028	0
df9926f0-b72f-40c8-aacb-371f480c382d_Basecall_Alignment_templat-snap.1|AT5G03555|no_kog_value	FNA.6694	622	88.585	0
971fdfba-84d3-4f2f-9694-686640029e79_Basecall_Alignment_templat-snap.2|AT5G24010|no_kog_value	FNA.7313	622	87.942	0
080daa6c-7fe2-48ea-87c4-c9517fa59565_Basecall_Alignment_templat-snap.1|AT3G56040|no_kog_value	FNA.7095	606	88.449	0
bc1caee6-25c8-4eab-91a1-ab7862ffc422_Basecall_Alignment_templat-snap.1|AT2G16630|no_kog_value	FNA.6820	599	90.317	0
a404731c-5e35-4224-a7cc-b6a8bf64335c_Basecall_Alignment_templat-snap.2|AT4G17098|no_kog_value	FNA.6069	589	88.115	0
556e0add-73c8-423c-a509-d6a85c4e6cb4_Basecall_Alignment_templat-snap.2|AT5G66810|no_kog_value	FNA.6661	558	90.143	0
57e60607-09ce-4296-b94b-1aa52ce6d548_Basecall_Alignment_templat-snap.1|AT5G20600|no_kog_value	FNA.7009	513	89.474	1.92E-178
38b082ec-ba1d-46d7-b083-b0cea62d9618_Basecall_Alignment_templat-snap.1|AT2G05120|no_kog_value	FNA.5801	543	88.582	2.33E-178
be5017c8-7da1-4664-8669-bf2154902685_Basecall_Alignment_templat-snap.1|AT5G66550|no_kog_value	FNA.7865	557	87.792	1.64E-177
4f6b1f24-c53a-46dd-aa8d-b704bb34c980_Basecall_Alignment_templat-snap.1|AT1G77720|no_kog_value	FNA.5154	638	85.266	1.64E-172
07e27975-4d98-423f-8336-4ed15e7529d7_Basecall_Alignment_templat-snap.2|AT5G42400|no_kog_value	FNA.4685	745	82.819	2.46E-172
e7daf9df-94a3-4cdf-8871-b0661051cdbd_Basecall_Alignment_templat-snap.1|AT5G15400|no_kog_value	FNA.6240	874	80.778	9.38E-171
3fdb2a86-9d87-4719-ab0e-017e5455eb80_Basecall_Alignment_templat-snap.8|AT2G26470|no_kog_value	FNA.6207	771	82.231	1.20E-170
28bd5a56-84c2-45e4-ab0b-4aac306be8f0_Basecall_Alignment_templat-snap.1|AT5G39250|no_kog_value	FNA.7276	774	82.171	1.26E-169
4d5d8526-bee0-42bc-8b44-e40de8dd11dd_Basecall_Alignment_templat-snap.2|AT3G02690|no_kog_value	FNA.7186	610	84.59	9.26E-158
8615b335-8244-46ab-88ea-86729453a752_Basecall_Alignment_templat-snap.1|AT1G03600|no_kog_value	FNA.7396	539	86.085	3.18E-155
02bdfc7f-8c93-485e-9763-64cbfb4e6f21_Basecall_Alignment_templat-snap.2|AT5G52810|no_kog_value	FNA.7115	695	81.583	1.28E-148
35997ccb-b1f7-4754-95db-4aed232a1673_Basecall_Alignment_templat-snap.1|AT4G16970|no_kog_value	FNA.6779	573	84.119	1.33E-145
bced7ee0-67ec-4fd4-ae3d-d3b956d4afaa_Basecall_Alignment_templat-snap.2|AT1G31780|no_kog_value	FNA.6900	522	85.824	2.71E-145

MinION read	Wickett *et al.* (2014) alignment	BLASTN assignment		
		Length	% idents	*E*-value
d8704bfc-bab2-45ac-9c3b-f26861a03f15_Basecall_Alignment_templat-snap.6|AT5G17690|no_kog_value	FNA.5588	782	79.795	1.63E-144
82216d1a-e320-4d9c-9d76-965aa1e237dc_Basecall_Alignment_templat-snap.3|AT1G51405|no_kog_value	FNA.6950	505	85.941	2.76E-141
bf0b43e8-bbf7-43b5-b3c4-cd226d590786_Basecall_Alignment_templat-snap.1|AT5G07400|no_kog_value	FNA.6737	556	83.993	2.68E-135
1984cd8f-47a6-4238-854e-5a07a81263ab_Basecall_Alignment_templat-snap.2|AT1G01180|no_kog_value	FNA.7667	562	82.918	1.26E-129
c94f0c64-415e-4fec-856c-3bcc08ff8bb9_Basecall_Alignment_templat-snap.3|AT5G18200|no_kog_value	FNA.6646	523	83.365	2.07E-123
5ef0c980-cf8a-4a16-80ac-dc0862b24e5a_Basecall_Alignment_templat-snap.2|AT1G49980|no_kog_value	FNA.5986	556	81.835	2.05E-118
95a1791d-6956-49dc-ba47-43d40db6a68c_Basecall_Alignment_templat-snap.2|AT1G24460|no_kog_value	FNA.7487	617	80.713	6.15E-118
b07fd070-5e4e-4084-b045-84b7bfdaa5b8_Basecall_Alignment_templat-snap.1|AT5G45660|no_kog_value	FNA.7767	503	82.704	7.63E-113
b0f941df-f2b5-4641-bbf4-ea481792474f_Basecall_Alignment_templat-snap.5|AT5G41270|no_kog_value	FNA.6353	549	81.421	1.20E-110
6734b3c0-753d-4735-a0e4-eb09bd56fc8c_Basecall_Alignment_templat-snap.2|AT4G33160|no_kog_value	FNA.6322	666	78.679	5.75E-110
e0fe2314-70dd-4b02-9c58-b7a58c60d0f2_Basecall_Alignment_templat-snap.2|AT3G09250|no_kog_value	FNA.6193	565	80.177	1.34E-100

Supplementary Table 1 | *ab initio* gene models used in phylogenomic analyses. Individual reads containing *ab initio* predicted coding sequences, and corresponding mappings to Wickett *et al.* (2014) alignments (based on 1:1 reciprocal best-hit assignment via BLASTN, default parameters).

Supplementary Discussion

Rapid generation of data. Modelling of accumulation curves in simulated species identification by resampling empirical data (see Methods and Figure 3) shows that the precision of each-way BLAST hit biases rapidly stabilises once reads on the order of 10^3 – 10^4 have been processed. More simply, a putative species assignment for an unknown sample using RTnS data in *Arabidopsis* would be unlikely to change once this many reads have been sequenced and processed. In our experiment, sample DNA extraction and library preparation combined took around two or fewer hours in every case, and bioinformatic processing took place continuously (using a simple script, executing every five minutes, which completed analysis of each batch of new reads in less than a minute). We suggest that the key performance characteristic for field-sequencing (where species ID is the goal) is therefore peak yield-rate (long reads / unit time), not aggregate yield (total base-pairs sequenced).

In our experiment, the R9 chemistry available at the time (May 2016) gave peak yield-rates in excess of 10,000 reads/hour, more than half of which were long enough for the ID procedures outlined above. It is therefore tractable to ID species in three hours or fewer from tissue collection to identification.

We further suggest that similar behaviour could also be expected for any eukaryotic whole-genome shotgun dataset with similar length distribution characteristics to our data (N50 ~4,400bp) and similar target species divergence (up to 13Mya: Beilstein *et al.* (2010) *PNAS* **107**:18724-8, doi:10.1073/pnas.0909766107; with neutral substitution rates of the order of 10^{-9} substitutions.site^{-1}.year^{-1}: Kagale *et al.* (2014) *The Plant Cell* **26**:2777 doi:10.1105/tpc.114.126391). These examples concern each-way (reciprocal) ID, where discrimination between two species is required. For cases with three or more target species, comparison using a scalar bias statistic becomes more problematic. However, we note that cumulative bias accumulation curves (see Figure 3d) could be plotted for multiple comparisons. Here the slope parameters would provide a fast, simple and useful way to determine which species database comparison was most likely.

Supplementary Notes

Supplementary Note 1 | Illumina MiSeq sequencing runs. 300bp paired-end libraries were prepared for four samples using the same field-extracted DNA as used for MinION sequencing (AL2a and AT1a), according to the manufacturer's (Illumina, Inc.) standard protocol. Samples AL1a and AL2a (*A. lyrata*) yielded 8,143,010 and 7,048,060 paired reads, respectively, for total yields of 2,451,046,010 base-pairs (bp) and 2,121,466,060bp. Samples AT1a and AT2a (*A. thaliana*) yielded 8,924,824 and 8,033,488 paired reads, respectively, for total yields of 2,686,372,024bp and 2,418,079,8880bp.

Supplementary Note 2 | Whole-genome shotgun coverage and mapping to reference genomes. In total, 2.4Gbp of paired NGS reads were sequenced each for *A. thaliana* and *A. lyrata*, equating to approximately 20.2x and 11.8x coverage, respectively, of the available reference genome lengths. Amongst reads that could be directly mapped to these genomes with BWA, average read depths were 19.5x and 13.8x respectively (for *A. lyrata*; 14.9x for *A. lyrata ssp. petraea*).

In total (combining R7.3 and R9 reads, but excluding phage lambda DNA filtered using BLASTN) 240Mbp of data were RTnS-sequenced for *A. thaliana*, and 62Mbp for *A. lyrata*. These yields equate to approximately 2.01x and 0.3x coverage, respectively, of the available reference genome lengths. Amongst reads that could be directly mapped to these genomes with BWA, average read depths were 1.82x and 4.07x respectively (for *A. lyrata*; 4.3x for *A. lyrata ssp. petraea*). Using LAST, RTnS *A. thaliana* reads aligned to 54.7Mbp of the TAIR10 genome (46% of the total genome length) with an average nominal alignment accuracy of 78.4%. Similar (77% accuracy was observed amongst RTnS *A. lyrata* reads mapped with LAST to the *A. lyrata* or *A. lyrata ssp. petraea* draft genomes, though many fewer reads could be mapped (0.9Mbp).
